# Towards the development of foods 3D printer: Trends and technologies for foods printing

**DOI:** 10.1016/j.heliyon.2024.e33882

**Published:** 2024-06-29

**Authors:** Husam A. Neamah, Joseph Tandio

**Affiliations:** a Department of Electrical and Mechatronics Engineering, University of Debrecen, Debrecen, 4028, Hungary; b Technical Engineering College, Al-Ayen University, Thi-Qar, 64001, Iraq; c Department of Business Management, Al-imam University College, Balad, Iraq; d Mechatronic Systems Design, Eindhoven University of Technology, Eindhoven, 5612, Netherlands

**Keywords:** Food's 3D printing, Additive manufacturing, Extrusion-based 3D printing, Food ink

## Abstract

3D printing of food materials is among the innovations that could revolutionize people's food choices and consumption. Food innovation and production have advanced considerably in recent years and its market has shown rapid annual expansion. Printing food technologies are considered as a potential solution for producing customized foods such as military food, and astronaut food. The printable food ink material still lacks standardization and superior extrusion process compared to other 3D printing industries. This review paper aimed to provide a comprehensive review of the current foods 3D printing and the latest technology in certain terms and with its concrete applications. In particular, the following issues are discussed: the printing techniques, exudations classes, business prospects, technologies, printing parameters, food materials, safety, and challenges and limitations of food 3D printing along with possible improvement recommendations. Significant printing parameters have been summarized and the safety of the food printing has been evaluated. Moreover, this article also contains a detailed, tabular evaluation of technical approaches employed across researched based and commercially available systems. One of the major limitations that need to be resolved was standardization of food printing safety.

## 1 Introduction

Recent innovations in manufacturing have made 3D printers more feasible for enthusiasts and business owners. Commonly, 3D printers feed on plastic, but with the recent advacement now they can print metals, ceramics, and food [[1], [2], [3]]. The benefits of using 3D printing, in general, are flexibility in design, the accuracy of the product, minimum waste, material customization, and less human interaction [[4], [5], [6]]. In general, the 3D printing method is adding layer by layer of materials in predetermined routes generated by g-code. To get the g-code, a slicer software will “slice” 3D model into 2D layers and generate the construction pattern needed to print the model. Exoskeleton-based robots, a subset of assistive robotics, have been used in rehabilitation systems became more feasible with the help of 3D printing technology [7]. Furthermore, 3D printers have contributed to the acceleration and improvement of concept proofing, prototyping, and manufacturing processes in a multitude of sectors, including aviation, biological materials in medical care [8], automotive, and the electronic industries, as well as the construction industries [9].

In food 3D printing, instead of non-edible materials, the filament uses a formalized food ink [10], the ink is inserted into the extruder. To print the food, the extruder forces the ink, either viscous or watery [11], into the nozzle [12]. The actuators will move the nozzle in each direction to create the specific paths [12]. Important parameters required for successful printing include but not limited to temperature, extrusion rate, nozzle height, diameter of the nozzle, extruder moving speed, and slicer software [3,[13], [14], [15]]. After the food has been printed, sometimes for certain types of food a post-processing (baking, frying, toasting, steaming, etc.) is necessary [16,17].

During the few years, cake, which consists of starch, sugar, corn syrup, yeast, and cake frosting, chocolate, cheese, mashed potato, dough, ice cream, peanut butter, and blends of fruits and vegetables have been successfully printed [3,16,[18], [19], [20], [21], [22]]. Some researchers have begun to look for unusual food ink such as insects, algae, bacteria, and fungi [4,16]. Today, chicken, turkey, fish surimi, scallop, shrimp, and even beef have also been printed [4,4,[23], [24], [25]]. The critical variables for all types of food to be printed lies on its rheological and physical properties [12,[26], [27], [28]]. Since many foods have unprintable properties, some additives/flow enhancers are added to the food [4,12,29]. By altering its properties, the foods printability has been improved.

Recent advances also add a new dimension from 3D to 4D printing by inserting smart materials that can change color or shape when stimulated [16,23,30]. Some of the applications of 4D printing are identification of product decomposition by changing color [16,31], smaller packaging [32], better control of dimensional stability after dehydrated [15], and alteration of structure for artificial tissues [33]. The procedure of 4D printing is more complicated compared to 3D printing, because of the additional stimulant and software for simulation [15,30,34]. As a result, the shape distortion need to be planned first for the product to conform to desired result [35]. Because of the added dimension, even with the same model, the printed food may show different form with altering the stimulation process [36]. Lately, the integration of AI with 3D food printing is transforming the food industry by enabling the production of personalized, nutritious, and sustainable food products [[37], [38], [39]]. AI-driven innovations in food design, process optimization, and quality control are paving the way for a future where food is tailored to individual needs and produced efficiently and sustainably [40].

In recent years, food 3D printing has advanced significantly, and more sophisticated techniques have been discovered [41]. However, for engineers and technicians unfamiliar with the terminology, understanding the current state of technology can be challenging and time-consuming. This review aims to provide an inclusive and accessible overview of the current state and future prospects of food 3D printing technology. Which provides, technical and clear guidelines, towards the development of new foods 3D printers. Some of the review papers related to food 3D printing are summarized as Table 1. Mostly the review papers recapitulated the printing techniques, parameters, ink materials, fluid properties, and applications of the 3D food printing. In this review, the current state of 3D food printing technology is comprehensively analyzed, highlighting its unique trends and technological advancements. Various 3D printing techniques are classified based on their operational principles. Additionally, the safety considerations in food 3D printing are occasionally discussed, This review investigate and address these gaps by offering a thorough analysis of safety and hygienic standards, regulatory frameworks, and the integration of advanced computational methods to optimize the 3D printing process, as well as highlight the potential of Artificial intelligence in the foods 3D printing industry. The lack of comprehensive studies on the impact of additives on industrialization and food production, along with the examination of existing challenges, motivated the undertaking of this review.

**Table 1:** Summarized review articles in food 3D printing technology.

Aspect	Printing Techniques and Methods	Ink Materials	Safety	Business Analysis and Practical Applications
**Printing Techniques and Methods**	[16,42], [18,28,29,43,44]	[4,11,41,45]	[9,13,[46], [47], [48], [49], [50], [51], [52]]	[3,6,35,41,53]
**Ink Materials**	[4,8,41,45]	[4,41,45,54]	[9,13,[46], [47], [48], [49], [50], [51]]	[15,17,23,32,[55], [56], [57], [58]]
**Safety**	[9,12,13,17,[46], [47], [48], [49], [50], [51]]	[9,13,[46], [47], [48], [49], [50], [51]]	[9,12,13,17,[46], [47], [48], [49], [50], [51]]	[9,13,[46], [47], [48], [49], [50], [51]]
**Business Analysis and Practical Applications**	[16,42], [18,28,29]	[4,41,45,59]	[9,13,[46], [47], [48], [49], [50], [51]]	[3,6,35,41,53]

The paper is structured into several key sections. It begins with the 1. Introduction, setting the context and importance of 3D food printing technology. The 2. Method section details the methodical review approach used in the study. This is followed by an analysis of the 3. Business and Research Publication Trend on Food 3D Printing, highlighting market growth and research interest. The paper then delves into 4. Printing Techniques for foods printing, with a detailed look at 4.1 Extrusion for foods printing technologies. Next, 5. Several Applications of Food 3D Printing are discussed, showcasing various practical uses. The section on 6. Commercial foods 3D Printer explores different commercial printers available in the market. 7. Notable Mention of Recent Experimentation highlights recent research advancements. The integration of 8. Artificial Intelligence Linked to 3D Printing and Trends in Personalized Nutrition is examined, followed by a discussion on 9. Hygienic Requirements and Food Ink Characteristic. The 10. Categories of Food Ink and Hygiene section categorizes various food inks and their safety. The role of 11. Additives/Flow Enhancers in improving printability is explained. The paper concludes by addressing 12. Challenge and Limitation of current technologies and providing a 13. Conclusion summarizing the findings. Finally, a comprehensive list of References is provided.

## 2 Method

An organized review following the PRISMA methodology was conducted to describe the current 3D printing techniques applied to design food materials. The techniques were classified based on material supply: liquid, powder, and cell culture. The review included searches in ScienceDirect, PubMed, Web of Science, and Scopus using specific keywords related to “food 3D printing, additive manufacturing, food ink, digital gastronomy, food customization, printable food material, space foods, and digitalized food” [60,61]. The review process, conducted by Two authors to minimize bias, involved defining inclusion and exclusion criteria, and extracting data. High-quality publications from 2018 to 2024 were considered to provide a comprehensive view of recent advances. Exclusions were made for non-English publications, books and book chapters, and duplicates. Not related topics such as animal research, alternative techniques, clinical research, pre- and post-processing, drug applications, and packaging. Inclusion Criteria were made for only original research that examined the literature on 3D printing which Identifies essential ideas, characteristics, applications, materials, mechanics, and designs. The objectives of this review were to analyze the literature on 3D printing processes, identify essential ideas, characteristics, applications, materials, mechanics, and designs, and uncover cutting-edge advances, including new materials and groundbreaking advancements. The selected articles were thoroughly analyzed to understand the interactive factors essential for the rational choice of 3D printing techniques in food design, namely printability, applicability, and postprocessing feasibility.

The initial search yielded 446 articles, 25 duplicate items were removed, and 270 items were excluded through automatic database filtration. Four articles were excluded based on language criteria, Furthermore, 248 articles were excluded due to retrieval issues, and 56 articles were excluded after title and abstract examination for not meeting the inclusion criteria. The full texts of the remaining 102 papers were reviewed, focusing on high-quality papers relevant to the identified topic. Finaly, 41 articles were selected for the methodical review.

In summary, this methodical evaluation offers a detailed examination of 3D food printing techniques and their applications, ensuring a rigorous and unbiased review process to highlight significant research and developments in the field.

## 3 Business and research publication trend on food 3D printing

**Fig. 1:**
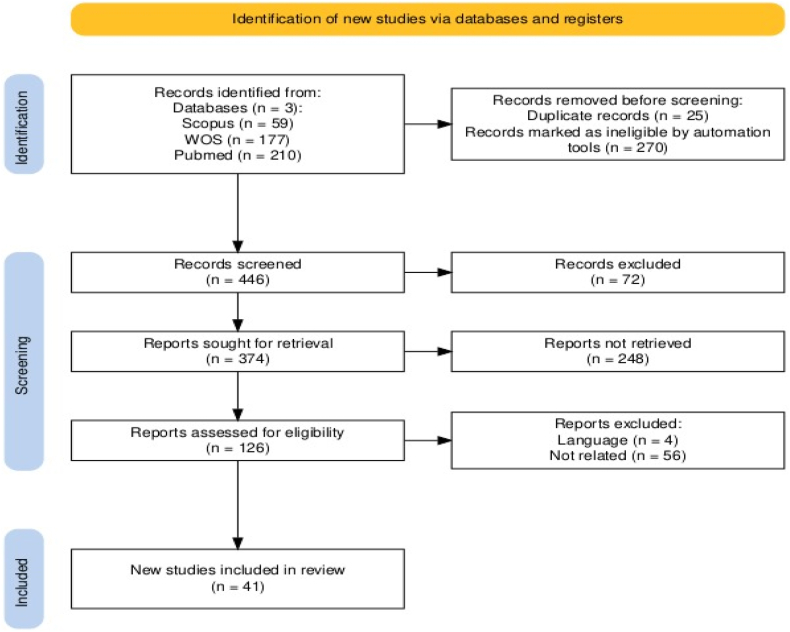
The updated PRISMA 2020 framework [67]: PRISMA flow diagram of this review study.

**Fig. 2:**
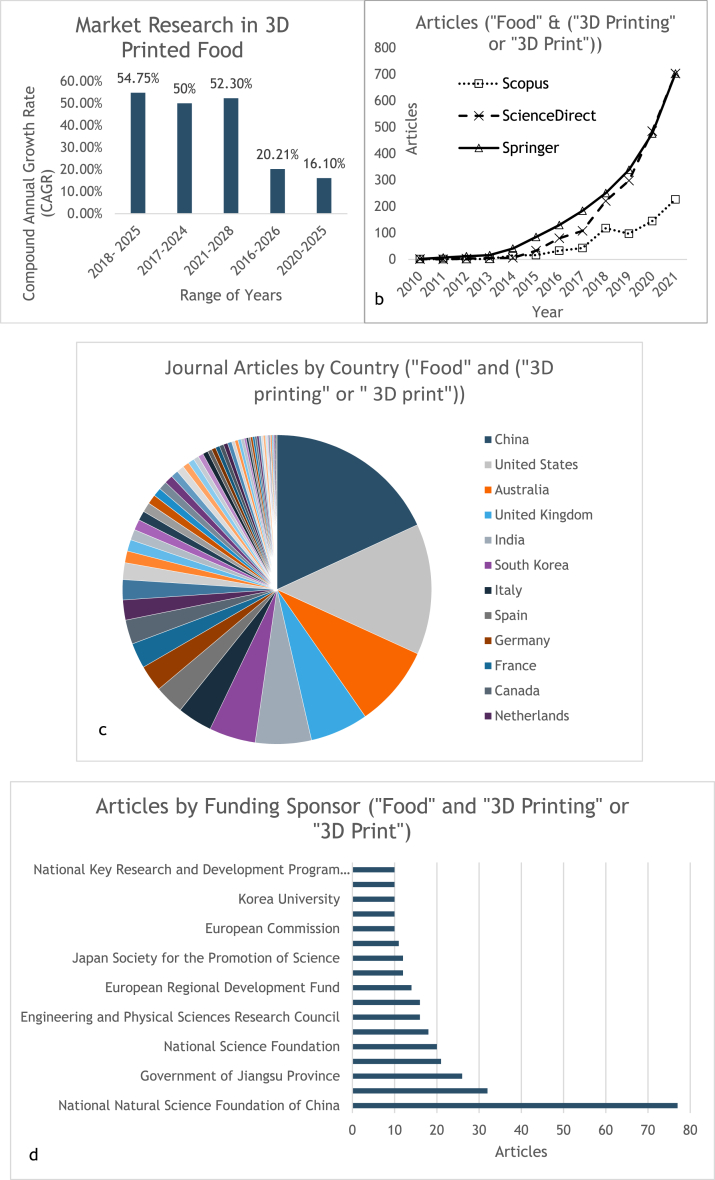
a. Market research conducted by several organizations. b. Number of articles about food 3D printing from 2017 to 2021 from Science Direct, Springer, and Scopus. c. Number of articles by country and d. funding sponsor from 2010 to 2022 based on Scopus.

Great business opportunities and high capital for food 3D printing has been progressing throughout the last years (see Fig. 1). The economic scale of food 3D printing may soar to $ 42.5 million by 2025 and the compound annual growth rate (CAGR) in 2018 is about 54.75 % [15]. Others calculated the CAGR for 2016–2026, 2017–2024, 2020–2025, 2021–2028 about 20.21 %, 50 %, 16.1 %, 52.30 %, respectively [32,[62], [63], [64]] as shown in Fig. 2a. Due to its advantages, the market may reach $ 1 billion by 2027 [23]. Businesses that may consider food 3D printer are restaurants, space companies, confectioneries, bakeries, and hospitals [65,66].

Although many people are unfamiliar with the use of 3D printers in food sector, few companies have tried to apply it in their business. An Italian startup, BluRhapsody, made pasta with different shapes using 3D printer [32]. Another company from Barcelona also used 3D printing to create meat imitation from plant [32,68]. An interesting company called REM3DY Health made tailored supplements and medicines for adults and children, and now moving toward protein bars [69]. In Japan, a startup called Open Meals printed “8-bit sushi” that is customized based on customers’ genomic and biometric data [70]. Mostly, the food 3D printing are in Europe and North America [32].

Interest in food 3D printing is increasing year by year. From *Science Direct* website, the number of articles is increasing year by year, as shown in Fig. 2b. The increases between each year are 169, 254, 285, and 452 from 2017 to 2021. Based on *Springer Link*, food 3D printing topic also raised from year by year as shown in Fig. 2b. Similarly, a search has been made for food 3D printing from *Scopus* website, and the results were showed in Fig. 2b–d. For now, China was the major investor in food 3D printing. From survey conducted, about 72 % is open to the idea of food from 3D printer [71]. This signifies that food 3D printing has great chance to create better opportunities in many sectors.

## 4 Printing techniques for foods printing

**Fig. 3:**
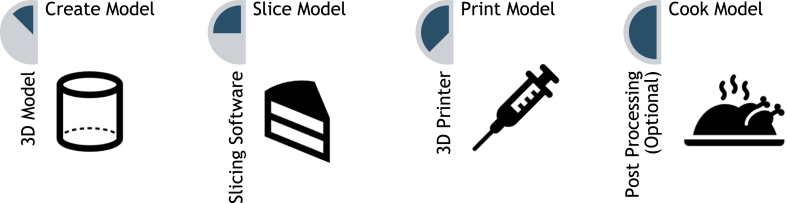
General steps of 3D printed food.

The general steps for food 3D printing are shown Fig. 3. Currently, there were many types of printing techniques for food printing [72,73], but in this paper only the main 4 types were emphasized. They were extrusion, selective sintering, binder jetting, and inkjet printing [16,58]. All the techniques had their advantages and disadvantages, therefore, to choose which technique to use was based on its application, material, and financial circumstances. Consideration of parameters [44] for extrusion printing need to be reviewed first to print successfully. The material properties, printing parameters, and extrusion mechanisms affect the printing result [16,18].

From some articles and books, the significant printing parameters of each printing method were summarized in Table 2. Key Printing Parameters and Their Effects in Food 3D Printing. This table includes parameters for extrusion-based, inkjet-based, and binder jetting techniques, detailing critical thresholds and their impacts on print quality. Although some printing parameters have been proved to cause an effect, to the extent of authors’ knowledge no study has showed how significant one parameter from the other statistically.

**Table 2:** Printing parameters of food 3D printing.

Printing Techniques	Printing Parameters	Equation	Effect	Ref
**Extrusion-based**	Nozzle height (or layer height), *h*_*N*_ (mm)	$h_{N\;critical}=\frac{1000Q}{v_{n}∙D_{N}^{2}}$ *Q* = material flow rate (mm^3^/s)	*h*_*N*_ < *h*_*N critical*_ à thick extruded line *h*_*N*_ > *h*_*N critical*_ à inaccurate deposition	[14,55]
**Extrusion-based**	Nozzle diameter, *D*_*N*_ (mm)	$h_{N\;critical}=D_{N\;critical}$	*D*_*N*_ < *D*_*N critical*_ à inconsistent extrusion *D*_*N*_ > *D*_*N critical*_ à lower resolution and accuracy	[12,14]
**Extrusion-based**	Nozzle speed, *v*_*N*_ (mm/s)	$v_{N\;critical}=\frac{4000Q}{\pi D_{N}^{2}}$	*v*_*N*_ < *v*_*N critical*_ à wide ink bead deposition *v*_*N*_ > *v*_*N critical*_ à narrow ink bead deposition *v*_*N*_ ≪ *v*_*N critical*_ à produce wavy lines. *v*_*N*_ ≫ *v*_*N critical*_ à interspaces in the lines	[14,74]
**Extrusion-based**	Printing temperature, *T*_*p*_ (°C)	NaN	*T*_*p*_ ↑ à (may) decrease viscosity. *T*_*p*_ ↓ à (may) increase viscosity	[12]
**Extrusion-based**	Infill density, *I*_*d*_ (%)	NaN	*I*_*d*_ ↑ à crispness and gumminess decrease, printing time and mechanical strength increase. *I*_*d*_ ↓ à the inverse effect of increasing infill density.	[75]
**Inkjet-based**	Droplet Size, $d_{D}\left(\mu m\right)$	$d_{D}\;\text{optimal}=f\left(P,\eta ,\gamma \right)$	- Optimal droplet size improves resolution and accuracy<br>− Too large or too small droplets can cause clogging or poor deposition.	[76,77]
**Inkjet-based**	Printing Speed, uP(mm/s)	N/A	-uP↑: Faster production but potential for lower resolution <br>− uP ↓ Higher resolution but slower production.	[78]
**Inkjet-based**	Substrate Temperature, Ts(°C)	N/A	−Ts↑: Can enhance droplet spreading and adhesion <br>− Ts↓: May lead to poor adhesion and spreading.	[79]
**Binder Jetting**	Binder Saturation, $S_{b}\;\left(\%\right)$	Sboptimal=f(Pb,ηb,γb)	- Optimal saturation improves binding and structural integrity <br>− Over-saturation can cause excessive binder use and weak structures.	[80]
**Binder Jetting**	Layer Thickness, $h_{L}\left(mm\right)$	N/A	- $h_{L}$ ↑: Faster build times but lower resolution <br>− $h_{L}$↓: Higher resolution but slower build times.	[16]
**Binder Jetting**	Curing Temperature, $T_{c}\left({}^{\circ}C\right)$	N/A	- $T_{c}$ ↑: Faster curing and better mechanical properties <br>− $T_{c}$ ↓: Slower curing, potential for weaker structures.	[4,81]

### 4.1 Extrusion for foods printing technologies

**Fig. 4:**
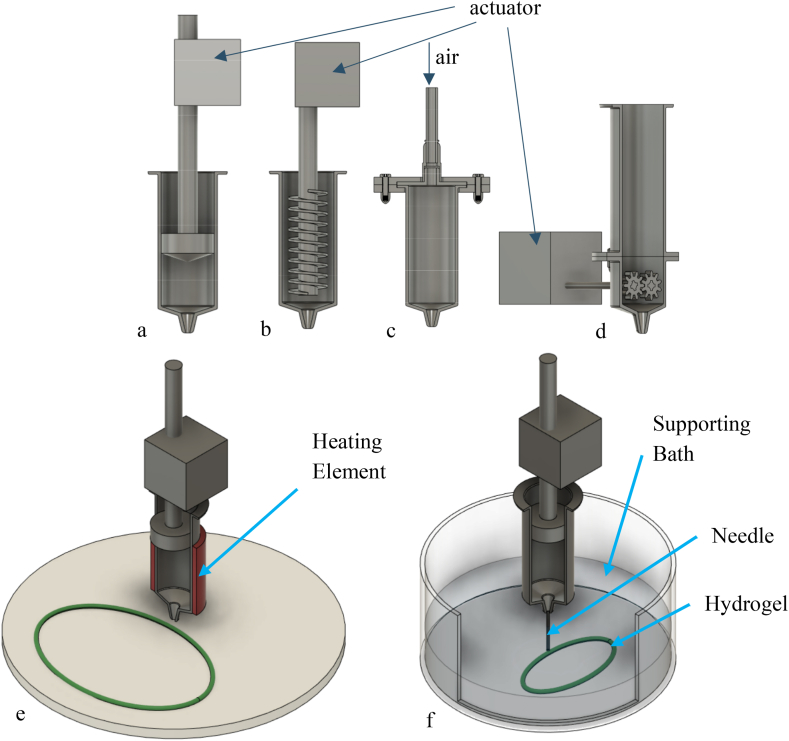
Extrusion-based 3D printing: a. Syringe-based, b. Screw-based, c. Air pressure-based, d. Gear-based; e. Hot-melt extrusion, f. Hydrogel-forming extrusion.

The most common printing techniques for food is the extrusion-based [13,28,82]. The process of extrusion is to force the food ink into the nozzle [55]. Its parameters are controlled digitally for precision and accuracy [83]. The four mechanisms are syringe-based, screw-based, air pressure-based, and gear-based [13,84] as shown in Fig. 4a–d. There are some advantages and disadvantages to the extrusion printing. Some of the advantages are easy adjustment of extrusion rate, easy development, wide range of printable food, high repeatability even with complex shape, low maintenance cost, and compact size [16,17,83]. The disadvantages are long fabrication time, anisotropic, vulnerable to warping and distortion, low level of precision [14,85]. These drawbacks can be overcome by changing the printing parameters and material properties.

The process of **syringe-based extrusion** (a) is quite simple. Food ink was inserted into the barrel, and an actuator will move the plunger to force the ink into the nozzle. To reduce the power of the motor, the syringe unit need to be carefully selected [13]. It should be noted that syringe-based extrusion is not a continuous process [14]. The syringe-based extrusion is used usually for high viscous food ink, and the extruded part is assumed to be able to support itself [16]. The configuration of this extrusion required one actuator for one printhead [13]. The method of the **screw-based extrusion** (b) is like the injection unit of injection moulding. Food ink was feed to the hopper, and the screw will rotate to force the ink into the nozzle [14]. For now, the screw-based extrusion is the only continuous printing method in the extrusion process. On the other hand, this method is limited to material with low viscosity [83]. The mechanism of **air pressure-based extrusion** (c) is very similar to syringe-based extrusion [83]. Instead of motor, the actuator use pneumatic pump to force the food ink [47]. The advantages of this extrusion are contamination of food is less and the weight of the printhead can be reduced since one pump can be used for multiple extruders [86]. The downside are the slow response time in changing the extrusion rate, need of air filtration, and additional devices to remove air bubbles when fill/refill in industrial scale [13]. a dual-extruder for food 3D printer based on selective compliance assembly robot arm and printing of various inks were presented [87]. The last **extrusion process is using gears** (d). Food entered from the hopper are forced out by two gears rotating reversely at a controlled temperature [84]. This mechanism is designed for food materials that are liquid at high temperature and solidify in a short time if the temperature drop [84]. Since this mechanism is quite new, there are only a few study that discuss this mechanism.

#### 4.1.1 Extrusion temperature

Besides, the mechanisms, the extrusion process is also divided according to its temperature. There are 3 classifications: “Room temperature extrusion” (RTE), “Hot-melt extrusion (HME)”, and “Hydrogel-forming extrusion (HFE)” [16]. The viscosity of the materials used in RTE must be considered to be sufficient for extrusion and rigidity [16]. The HME (Fig. 4e) needs materials that can be melted to liquid state and retain structural integrity after the extrusion. In the HFE (Fig. 4f), the hydrocolloid solutions or dispersions are extruded into a gel/polymer/supporting bath with jet cutter, syringe pipette, vibrating nozzle, and etc. [13]. After the deposition, the molecules began to crosslink–e.g., in the presence of calcium cation [33,47].The supporting bath need to be viscoelastic first, and then became self-supporting gels for the following layers [13,20].

#### 4.1.2 Other printing methods

**Fig. 5:**
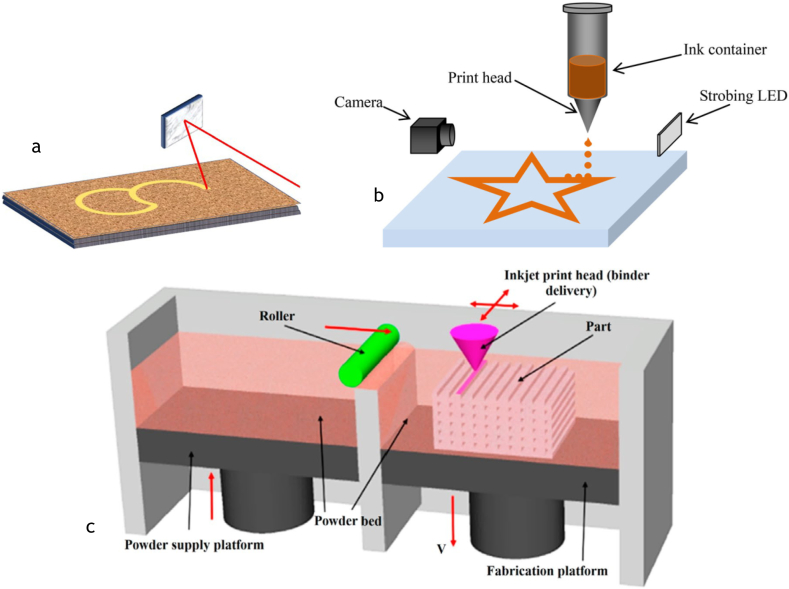
a. Selective Sintering. Adapted from Ref. [89], b. Inkjet printing. Adapted from Ref. [88], c. Binder Jetting. Adapted from [77].

**Selective sintering** (Fig. 5a) use the sintering source (laser/hot air) to choose which particle to be melted and fused together to form the final product [14]. After each scan of the sintering source, another layer of powder bed is added and the process is repeated until it finished [14,16,88]. Other method, called **binder jetting** (Fig. 5b) is like selective sintering. The only difference is a liquid binder is used to bind the layers, instead of sintering source [5]. After each scan, the supply platform will move upward, and the fabrication platform downward to allow the addition of powder [80,81]. The roller will transport the powder from supply platform to fabrication site, then the scanning process start again [16]. **Inkjet printing** operates like inkjet printer for paper. A stream of droplets are dispensed by the thermal or piezoelectric head into the food surfaces [55].

#### 4.1.3 Bio-printing

**Fig. 6:**
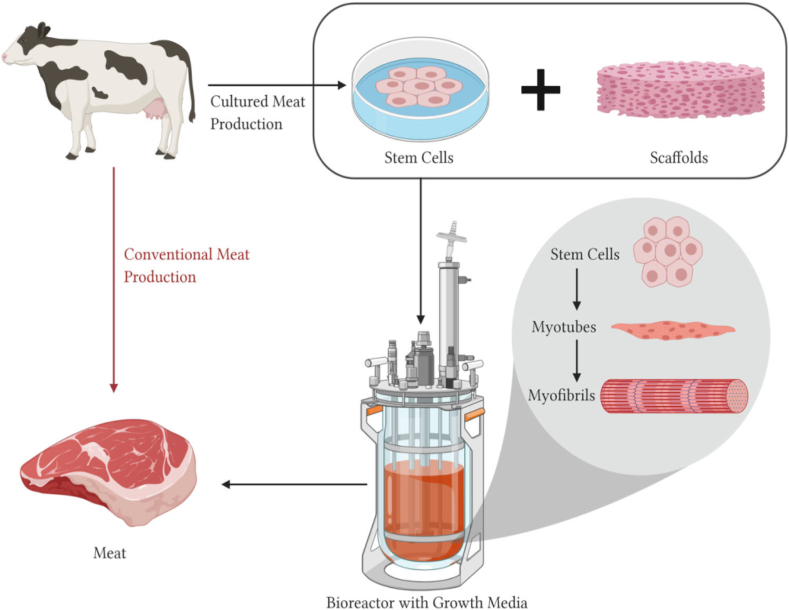
Cultured and conventional meat production process. Adapted from [56].

Bio-printing technique is based on tissue engineering [4], which is utilizing cells to develop food [50,90]. The process is done by depositing living cells with either a micro-extrusion, an inkjet printer, or laser-assisted printer into a support platform [46,49]. The cells will construct the tissue and the platform will be removed [4]. The tissue is placed in a bioreactor with a low frequency stimulation to maturate the fibers from stem cells to myotubes then to myofibrils [46,56,91]. Some difficulties of this method is cost-effectiveness, cell damages induced in the printing, size of construction, and the mechanical and structural [4,49]. The deposition of the ink may use inkjet [77,92], extrusion, SLS, stereolithography (SLA), and digital light process (DLP) [90,93]. The most common method is extrusion, because of its controllable shapes, pores, porosity, and cell distribution [56]. Some of the available starter cells that show good prospect are satellite stem cells and Adipose-derived stem cells (ADSCs), but further research needs to undergo for mass production. The growth medium commonly used fetal bovine serum (FBS) and antibiotics, but they aren't well accepted because of controversies [56]. The scaffolding can be made of porous materials using cellulose, fibrin, alginate, silk, chitosan, or collagen [49,56]. Hydrogels may be used instead of scaffolds [49]. The process of cultured meat production is shown in Fig. 6.

## 5 Several applications of food 3D printing

Interest in food 3D printing has increased much nowadays [3,5,16]. From space company to home users, they can benefits from this technology [56,88]. In addition, for people with specific needs, the food can be tailored according to their health requirements [16,48,55,94]. By using 3D printers, less waste are generated and cooking is more simple than before [1,5,13,94]. To simplify the process for new consumers of food 3D printing, it is possible to use e-recipes to print the food, therefore minimizing failure and managing nutrient content [46,58]. In addition, food packaging, edible or inedible, and the food may be printed directly [32,49]. A new development of combining the product and packaging into one unit also has been made by Mission Viejo in California [32,95].

One of the example of food 3d print in utilizing waste was done by Jagadiswaran and colleagues [96]. The food wastes, which are grape pomace and broken wheat, are transformed into cookies. These cookies have high proteins and dietary fiber. After baking, the cookies shape does not deform from its initial shape. The food 3d printer is a home-made fabricated extrusion printer. This may give proposition to business owners to reduce waste and consequently cost. From experiments conducted by various researchers as shown in Table 3 the printing parameters vary with each material. Therefore, the most common way to get acceptable parameters was by experiments. To aid the readers for quick review of the research articles, the table has been divided into 7 columns. By searching for the appropriate category, the readers can quickly find the relevant research articles.

**Table 3:** Research on food 3D printing by various researchers.

Temp.	Printing Method	Material	Stage of Development	Field of Application	Remarks	Ref
RTE	Screw-based extrusion	Lemon juice and potato starch	Laboratory stage	Gel production	The best proportion of potato starch in lemon juice was 15g/100g. Nozzle diameter of 1 mm, extruder rate of 24 mm^3^/s, and nozzle movement speed of 30 mm/s were the best parameters founded.	[19,97]
RTE	Syringe-based extrusion	Dried mushrooms, bananas, dried non-fat milk, white beans, and lemon juice	Laboratory stage	Children snack	The printed snack of fruit-based food was similar to the model. The flow affects filament thickness and pore diameter distribution, while print speed influences growth rate and pore diameter distribution.	[98]
Varies	Binder jetting (Fujifilm Dimatix ink jet printer)	Amorphous cellulose powder, food ink based on xanthan gum	Research stage	Food industry	The powder can be recrystallized by controlling moisture and temperature. The temperature plays a crucial role for the cohesion of the powder and the ink. The printed parts are still only 2D.	[5]
RTE	Syringe-based extrusion	Starch, cellulose nanofiber, oat protein concentrate (OPC), rye bran, milk powder, and faba bean protein concentrate (FBPC)	Laboratory stage	Healthy snack	Best material compositions are 10 % starch + 35 % OPC, 60 % semi-skimmed milk powder, 15 % skimmed milk powder, 30 % rye bran, or 45 % FBPC	[99]
RTE	Syringe-based extrusion	Carrots, pears, kiwi, broccoli rabe leaves, and avocado	Laboratory stage	Healthy snack	The process of printing did not change the taste, antioxidant capacity, and total phenolic content. There is a need of sanitizing the printer parts, because bacteria concentration of 4.28 Log CFU/g was found.	[76]
RTE	Screw-based extrusion	Surimi made of carp fillet and salt (NaCl) at different concentrations	Laboratory stage	Fish industry	Good printability is found with NaCl of 1.5g/100g. Gel strength, water holding capacity, and network structure increased as NaCl increases. Nozzle diameter of 2 mm and 5 mm nozzle height yield the optimal bonding.	[100]
RTE	Screw-based extrusion	Potato starch, pea protein, water, and butter	Laboratory stage	Food industry	To print potato starch, pea protein is necessary. The best compositions were 45 % potato starch, 1 % pea protein, 9 % butter, and 45 % water.	[101]
RTE	Coaxial syringe-based extrusion, syringe-based extrusion	Low methoxyl (LM) pectin and CaCl_2_	Laboratory stage	Bioprinting	Coaxial extrusion did not need post treatment to allow gelation of the pectin. There may be syneresis as time passes, which depends on Ca^2+^ concentration. Both method of extrusions results in good accuracy, but there is a need of further study.	[72,91,102]
RTE	Syringe-based extrusion	Orange fruit concentrate (OC), wheat starch (WS), xanthan gum (XG), k-carrageenan gum (KG), guar gam (GG), and gum arabic (GA)	Research stage	Fruit-based product	KG blend showed good structural integrity and loading bearing capacity. The best compositions are achieved by OC-WS-KG.	[103]
RTE	Syringe-based extrusion	Ketchup, chocolate pudding, peanut butter, mayonnaise, and jam	Research stage	Food and Medical industry	The extrusion is effective for water content higher than 33 wt%. Hagen Poisoulle equation can predict the actual discharge volume with 3 % error, but for material with high viscosity variation the error may reached 17 %.	[104]
RTE	Syringe-based extrusion	Flour, calcium caseinate, water	Laboratory stage	Dairy products	By expanding the surface-to-volume ratio, the probiotics concentration surpassed 10^6^ CFU/g when baked at 145 ^°^C.	[105]
NaN	NaN	cultured meat and insects	Case study	Food industry	In Australia, people were not open to the idea of cultured meat or insects printed with food 3D printer.	[106]
RTE	Syringe-based extrusion	Potato powder, whole milk, soybean lecithin, agar-agar, sodium alginate, and glyercol	Laboratory stage	Dysphagia patient	Additives that give better printing stability were sodium alginate and agar-agar. The best parameters of the 3D printer were 4 mm nozzle diameter and 5 mm nozzle height.	[19,107]
HME	Syringe-based extrusion	κ-carrageenan and water	Research stage	food and pharmaconutrition	Gel strength was inversely proportional to printing speed and layer height. The gel was successfully printed without adding additives.	[108]
Food 3D printer with IR heating	Syringe-based extrusion	Dough, jujube jam, sesame paste, shrimp paste, ground chicken,	Prototype	Food industry	The printer can print with great precision, and with the Infrared lamp, the part can be cooked directly.	[22,25]
RTE	Air-based extrusion	Wheat flour, mango powder, olive oil, and water	Laboratory stage	Food industry	The best compositions of flour-to-water, flour-water-olive oil (2 % w/w olive oil), and flour-water-olive oil-mango powder (2.5g mango) ratio are 5:3, 55:2.75:30, and 57.5:30:3:2.5, respectively.	[22]
RTE	Syringe-based dual extrusion	Lean beef paste, fat/lard	Laboratory stage	Food industry	Increasing fat content causes higher cohesiveness and shrinkage, and lower chewiness and moisture retention. Addition in infill density shows the opposite effect of increasing fat. The interaction of both also influences the properties, except fat retention and dimensional printing deviation.	[24]
RTE	Screw and syringe-based extrusion	Mashed potato, water	Research stage	3D printer company	Screw-based not suitable for high viscous ink. Syringe-based has simpler fluid characteristic compared to screw-based	[83]
RTE	Syringe-based extrusion	Anthocyanin-potato starch gel, lemon juice gel	Laboratory stage	Food industry	The printed anthocyanin-potato starch gel can successfully change its color over time or when sprayed with pH solutions.	[109,110]
RTE	Air-based extrusion	Green gram, fried gram, barnyard millet, and ajwain seeds	Prototype	Healthy snack	To preserve the nutrients with minimum color and textural lost, microwave drying was the best. All finished samples have acceptable taste.	[111]
HME	Syringe-based extrusion	Cordyceps flowers, vegetable oil, isomaltose, low-acyl kappa-carrageenan, agar (purity >98 %), low-acyl gellan gum	Laboratory stage	Food industry	The best ratio to print was found: powder: water:isomaltose:oil:agar = 10:10:3:1:0.1	[112]
HME	Syringe-based extrusion	Soy protein isolate (SPI), gelatin, sodium alginate, and water	Laboratory stage	Food and medical industry	SPI with additives showed shear-thinning behaviour. Mixtures of SPI and Gelatin (2, 6, and 10 %) have good printability.	[113]
NaN	NaN	NaN	Research stage	Restaurants	About half of the participants showed positive attitude toward food 3D printing. They believed that 3D printed food can improved today's problems.	[71]
RTE	Syringe-based extrusion	None	Prototype	Hobbyist	FDM plastic 3D printer can be modified to print food	[114]
Dual extrusion with near infrared heating	Progressive cavity pumps-based extrusion	Hydrated wheat starch, egg white powder, desalted egg white powder	Research stage	Food industry	3D SLTS (Structuring, taste Localization, Thermal Stabilization) technique empowers the repeatability of the textural setting in the production of starch-egg white powder systems. The concentration gradient of NaCl has been successfully manipulated. Comparable texture of different NaCl localizations were achieved.	[73]
RTE	Syringe-based extrusion	“Textured soybean protein” (TSP), “drawing soy protein” (DSP)	Prototype	Vegetarian food	Xanthan gum (an additive) and TSP showed the best printing characteristic and integrity after frying. The printed materials texture properties were like chicken breast.	[115,116]
HME	Syringe-based extrusion	sodium caseinate, cheese, chocolate, coconut butter	Prototype	3D printer company	By knowing the printing speed and pressure, and flow rate exponent and coefficient, the material extrudability can be predicted.	[117]
RTE	Syringe-based extrusion	Grape pomace, broken wheat	Case study	Food industry	Waste from food industry can be utilized for food ink.	[96]
RTE	Syringe-based extrusion	What flour	Research stage	Food industry	For a wheat flour dough, a G′ in the range 3–10 kPa and a tanδ in the range 0.15–0.17 shows good printability	[118]
HME	Syringe-based and gear-based extrusion	Syringe-based: sodium alginate, gelatin, common bean protein extract (CBPE), water Gear-based: agar, xanthan, CBPE, water	Laboratory stage	Food Industry	Superfine grinding on CBPE reduces printability for both extrusion methods. A type of protein is lost during the gear-based extrusion.	[84]
RTE	Air-based extrusion	Vegetables, chicken broth and thigh, olive oil, spices	Laboratory stage	Meat-based snack	Chicken-based snack was successfully 3D printed with the following parameters: 90 % feed rate, 110 % flow rate, 0.5 mm nozzle height, and 1.79 % gelatin concentration.	[119]
HME	Screw-based extrusion	Potato starch, pea protein, xylose with protein enzymatic hydrolysate, and glycerol	Laboratory stage	Food industry	By adding enzymatic hydrolysate (xMRPs), the 3D printed product has better fusion, concealed laminar boundaries, structure, and antioxidant activity. The best printing material was 6g xMRPs.	[120]
RTE	Syringe-based extrusion	Whey protein isolate (WPI), canola oil, water, and corn starch	Research stage	Food industry	Corn starch affects more storage modulus (*G′*) and loss modulus (*G″*) than WPI. Too much WPI decreases printability. Better printing can be achieved with low temperature.	[121]

The key conclusions that can be drawn about the state of research and development in food 3D printing are summarized in Table 3. The materials range from common food items like lemon juice and potato starch used in screw-based extrusion for gel production, to more complex formulations such as κ-carrageenan and water in syringe-based extrusion for food and pharmaceutical applications [19,97,98,104,108]. This variety in materials indicates the broad applicability of 3D printing in creating diverse food products, addressing specific dietary needs and preferences. The stages of development predominantly reflect laboratory research, with several methods having reached the prototype stage, such as the syringe-based extrusion of dough, jujube jam, and ground chicken, indicating a progression toward practical application in the food industry [22,24,[109], [110], [111]]. The practical implications are significant, as demonstrated by the food 3D printer with infrared heating, which allows the printed part to be cooked directly, showcasing the integration of printing and cooking processes [22,24]. Fields of application include the food and medical industries, with specific mentions of dysphagia patients benefiting from tailored nutritional profiles and textural properties of 3D printed foods, and the potential for bioprinting, highlighted by the coaxial syringe-based extrusion of low methoxyl pectin and CaCl₂ [19,107] [72,91,102]. This highlights the potential for 3D printing to address specific health and dietary needs, providing personalized nutrition solutions. Notably, the remarks section highlights significant findings, such as the impact of different hydrocolloids on printability and texture, where additives like sodium alginate and agar-agar improved the stability of printed potato powder and whole milk for dysphagia patients [19,107]. There is also mention of microbial safety concerns necessitating the sanitization of printer parts, due to bacterial concentrations found in printed foods, emphasizing the importance of hygiene in food 3D printing [76,100]. Innovative uses of 3D printing technologies are also noted, such as the ability to print different materials with milk ink at room temperature and the successful color-changing properties of printed anthocyanin-potato starch gels when exposed to pH changes [[109], [110], [111]]. These examples stage the technological advancements and potential for creative applications in food printing, driving the field forward with novel functionalities. Overall, these detailed observations underscore the varied and rapidly advancing nature of 3D food printing technologies. They point to a future where personalized nutrition, enhanced food safety, and sustainable food production are increasingly feasible, marking significant progress and potential in the field [96,97,[120], [121], [122]].

### 5.1 Commercial foods 3D printer

The market must assess the production rate of 3D printed foods (3DPFs) as demand continues to grow. The increased consumption of 3DPFs across various applications, such as food personalization, meeting specific nutritional needs, airline meals, hospital requirements, and space travel [66], has significantly expanded their market. By 2023, the 3DPF market is projected to reach $525.6 million, reflecting an annual growth rate of 46.1 %. This rapid increase underscores the escalating demand in global markets. Market research indicates that meat-based products constitute 7.1 % of the market, dairy products account for 16.1 %, dough products make up 22.4 %, and fruit and vegetable-based products represent 10.5 % [29,36,123]. Diverse range of commercial food 3D printers are summarized in Table 4., highlighting their applications, target users, and technological advancements. Mid to small-sized bakeries can enhance productivity and consistency using the 3D Dessert Decorator, while the Deco-Pod and Cake Writer™ by Beehex offer customizable decoration solutions for grocery stores, in-store bakeries, and direct contact stores, reducing labor and engaging customers through personalized designs [124], BeeHex created pizza printer with approximately 5 min faster processing time [13]. The Chef 3D printer caters to restaurants, enabling multi-layer pizza printing and customizable toppings [125]. ChefJet 3D Printer by 3D Systems and Brill 3D Culinary Studio target industrial kitchens and molecular gastronomists, supporting complex confectionery and cake decorations with high precision and versatile material support [[126], [127], [128]]. Choc Edge's Choc Creator V2.0 Plus and mycusini® 2.0 by mycusini offer chocolateries and home users professional-quality chocolate creations with fine detail and ease of use [129]. Chocola3d, suitable for educational institutions and cafes, supports a variety of materials, promoting creativity and learning in food technology [130]. Procusini® 5.0 and Focus 3D Food Printer by byFlow cater to professional settings like caterings and chocolateries, enhancing dessert offerings and on-site customization [[131], [132], [133]]. PancakeBot 2.0 and ZBOT's Commercial Art Pancakes Printer F5 attract customers with creative pancake designs, increasing efficiency in pancake houses [134,135]. FoodBot-S2 by Changxing Shiyin Technology Co., Ltd. and Foodini by Natural Machines expand automated and innovative food solutions for vending machines, retail, and educational institutions, supporting diverse materials from chocolate to mashed potatoes [[136], [137], [138]]. Finally, the Sweetin 3D Food Printer by Wiibox provides high-precision food preparation for restaurants and home users, facilitating creative culinary possibilities with multiple recipe support [139]. Other companies that made food 3D printers based on binder jetting are 3D systems and Fujifilm Dimatix and for inkjet printing are De Grood Innovations and TNO [85]. These developments demonstrate the versatility and technological impact of 3D food printing across various sectors, from small bakeries to industrial kitchens and home use.

**Table 4:** Commercial food 3D printers.

3D Printer	Organization	Products	End users	Product Features	Product image	Technological Impact	Ref
3D Dessert Decorato-r	Beehex	Decoration for cookie, cupcake, cake, etc.	Mid to small-sized bakeries	High precision, easy to use, fast setup		Increases productivity and consistency in small bakeries	[124]
Deco-Pod	Beehex	Decoration for cookie, cupcake, cake, etc.	Direct contact store (e.g., grocery store, bakeries)	Compact design, user-friendly interface, customizable designs		Enhances customer engagement through customization, reduces manual labor	
Cake Writer™	Beehex	Decoration for cookie, cupcake, cake, etc.	In-store bakeries	Fast printing speed, versatile decoration options, minimal training required		Reduces labor costs, increases product consistency and appeal	
Chef 3D	NA	Pizza	Restaurants	Multi-layer printing, customizable toppings, robust build		Revolutionizes restaurant food preparation, enables customized dining experiences	[125]
ChefJet 3D Printer	3D Systems	Confection, cake decoration	Industrial kitchen, mixologist, molecular gastronomists,	High-resolution printing, large build volume, supports multiple materials		Enables complex and high-quality food decorations, enhances culinary creativity	[126,127]
Brill 3D Culinary Studio				Precision printing, customizable recipes, durable construction		Supports innovative culinary techniques, improves efficiency in professional kitchens	
Choc Creator V2.0 Plus	Choc Edge	Couverture chocolate	Chocolateries	Fine detail printing, easy to clean, temperature control		Enhances chocolate craftsmanship, reduces production time	[128]
mycusini® 2.0	mycusini	Chocolate	Home-users, small chocolateries	Compact size, affordable, user-friendly software		Makes professional-quality chocolate creations accessible to home users	[129]
Chocola3d	Chocola3d	Chocolate, cheese, dough, vegetables, fruit pastes	Education institution, home-user, confectioneries, cafes	Versatile material compatibility, educational applications, reliable performance		Promotes learning and creativity in food technology, suitable for various settings	[130]
Procusini® 5.0 3D Food Printer	Procusini	Chocolate	Caterings, hotels, confectioneries, gastronomies, bakeries	High-quality output, multiple material support, user-friendly interface		Ideal for professional environments, enhances dessert and decoration offerings	[133]
Focus 3D Food Printer	byFlow	Chocolate, meringue, fruit, cookie, meat	Pasteries, chocolateries	Portable, versatile, high-resolution printing		Enables on-site food customization, broadens product offerings	[131,132]
Commercial Art Pancakes Printer F5	ZBOT	Pancake	Pancake house	Detailed pancake designs, fast operation, easy maintenance		Attracts customers with unique pancake art, increases efficiency	[135]
PancakeBot 2.0	PancakeBot	Pancake	Pancake house	Customizable designs, user-friendly, compact size		Offers creative breakfast options, engages customers	[134]
FoodBot-S2	Changxing Shiyin Technology Co., Ltd.	chocolate, biscuits, milk candy, pastries, jams, mashed potatoes	Vending machine, store, educational institution	Multi-material support, high precision, easy integration		Expands automated food offerings, suitable for various retail environments	[137,138]
Natural Machines Foodini	Natural Machines	Chocolate, cake, vegetables, cheese, cupcake, meat, cookies	Restaurants, hospitals, R&D companies, educational institutions	Versatile material use, intuitive interface, robust design		Supports innovative food solutions, enhances nutritional offerings	[136]
WiibooxSweetin 3D Food Printer	Wiiboox	Chocolate, mashed potato, fruit jam, biscuits, cream candy, bean paste	Restaurants, home-users	High precision, multiple recipe support, compact design		Facilitates creative food preparation at home and in restaurants, broadens culinary possibilities	[139]

## 6 Notable mention of recent experimentation

Th The notable recent experimentation in food 3D printing encompasses significant advancements that contribute uniquely to the field, each offering distinct technical insights and practical applications. Researchers from Singapore have achieved a milestone by 3D printing fresh and frozen vegetables tailored for dysphagia patients [122], his study focuses on raw vegetables like peas, carrots, and bok choy, enhanced with hydrocolloids such as xanthan gum (XG), kappa carrageenan (KC), and locust bean gum (LBG) to improve their rheological properties [27,28,91,102,140].

The vegetables were turned to puree by blending boiled peas and carrots, and steamed bok choy. Liquid from the purees was strained. Peas (high starch) with 80 % water content did not need HCs, carrot (medium starch) with 90 % water content needs a single HC (0.3 % w/w XG), and bok choy (low starch) with 96 % water content needs 2 HCs (1 % w/w XG, 2 % w/w LBG). Although the rheological, textural, printability, and microstructural properties have been analyzed, nutrients content of the 3D printed vegetables have not been conducted. Study related to development of High-hydration hydrogels based on carbohydrate polymers and green formulation methods have attracted intensive research focus recently. Driven by the attractive functions of starch, oxidized maize starch (OMS) was select and the associated hydrogel (3D-OMS) was fabricated by hot-extrusion 3D printing (HE-3DP) [110]. Meat analog production through artificial muscle fiber insertion using coaxial nozzle-assisted three-dimensional food printing [91].

Study related to 4D printing of mashed potato have been conducted by researchers from China [141]. The ingredients used were potato flakes, purple sweet potato powder, sodium alginate, citric acid, and sodium bicarbonate. The purple sweet potato powder was selected because of its anthocyanin component that can change colors when stimulated with acidic or basic solution. The inks for mashed potato are set by mixing specified weights of potato flakes with 2 % sodium alginate. Hot water is then added and stirred. The acidic and basic inks can be obtained by adding 1 % citric acid and 1 % sodium bicarbonate, respectively. The other inks for the coloring are prepared with purple sweet potato powder instead of potato flakes and no citric acid or sodium bicarbonate is added. The result showed that the rate of color change is inversely related to the potato flakes content, and after some time passes, acidic, neutral, and basic mash potato showed red, blue-purple, and green color, respectively. All the printed samples have acceptable taste.

Additionally, notable experiments include Revo Foods' launch of the first 3D-printed vegan salmon filet available in supermarkets, using their patented MassFormer technology [142]. This development highlights the capability to integrate fats into a fibrous protein matrix, achieving the texture and flakiness of fish filets, and demonstrates the potential for mass production of 3D-printed foods [143]. Researchers from Kazakhstan have also highlighted the potential of 3D printing to create plant-based alternatives using ingredients like soybean, pea, lentil, and buckwheat, which could help reduce environmental impacts and promote sustainable eating habits [144,145].

**Fig. 7:**
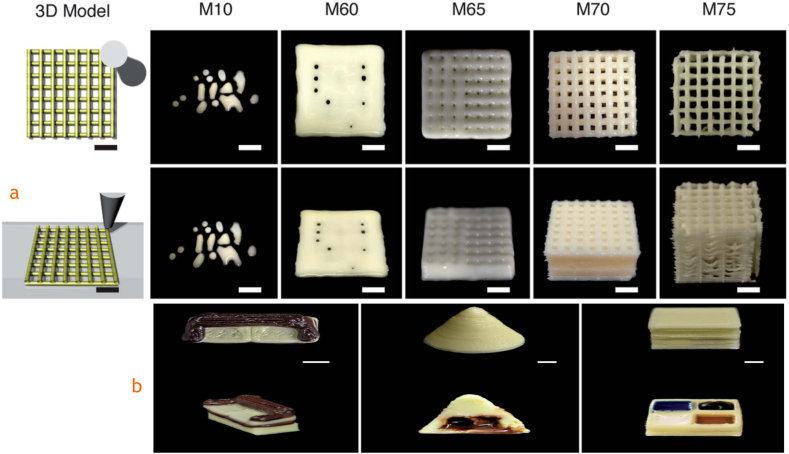
a. DIW 3D printed milk compared to the model. Adapted from Ref. [146], b. Left: DIW 3D printed milk and chocolate; Middle: DIW 3D printed milk with chocolate syrup; Right: DIW 3D printed milk with milk cream, maple syrup, blueberry syrup, and chocolate syrup. Adapted from [146].

Moreover, the study on direct ink writing (DIW) 3D printing of milk using cold extrusion, without additives, highlights the shear-thinning properties of milk ink [146]. The successful printing of milk with a 70–75 % w/w milk powder concentration demonstrates the potential to create dairy products with structural integrity at room temperature. The versatility of milk-based inks, shown through the incorporation of various syrups and creams, underscores the practical applications for diverse and customizable dairy products. The shear-thinning behavior, modeled by the Herschel-Bulkley equation (n < 1), ensures smooth extrusion and consistent quality, essential for scalable production. The printing results of the milk ink and with other materials are shown in Fig. 7a and b.

These developments are categorized as unique due to their pioneering approaches in addressing specific challenges and expanding the capabilities of 3D food printing. The Singapore study on vegetable printability enhances nutritional solutions for medical applications, providing a pathway for the creation of tailored dietary options for patients with specific health needs. The OMS-based hydrogels align with sustainability and health trends, offering an eco-friendly solution that maintains desirable textural properties. The 4D printing of mashed potatoes introduces dynamic, responsive food products that engage consumers through interactive experiences, showcasing the potential for culinary innovation. The DIW of milk demonstrates practical and versatile applications for dairy, highlighting the technology's capability to produce high-quality, customizable food products efficiently. Collectively, these advancements underscore the transformative potential of 3D food printing technology, reinforcing its significance and broadening its impact in both academic research and practical applications.

## 7 Artificial intelligence linked to 3D printing and trends in personalized nutrition

The integration of artificial intelligence (AI) with 3D printing technology is revolutionizing the food industry, particularly in the realm of personalized nutrition [147]. AI enhances 3D food printing by optimizing design, improving production efficiency, ensuring quality control, and enabling the customization of nutritional content. This synergy promises to deliver highly personalized and nutritious food products tailored to individual dietary needs and preferences. [40], food testing [38], and food safety [39,52]. AI technologies, such as machine learning (ML) and deep learning (DL) algorithms [148], play a crucial role in the pre-processing, processing, and post-processing stages of 3D food printing [37]. During pre-processing, AI aids in predicting the rheological properties of food inks, ensuring that the selected materials possess the necessary flowability, shear stability, and structural integrity for successful printing [39]. For instance, AI can analyze the complex interactions between ingredients like hydrocolloids and proteins to determine optimal formulations that enhance printability and final product quality [149]. During the printing process, AI algorithms can dynamically adjust parameters such as extrusion speed [44,44], nozzle temperature, and layer height to maintain consistent print quality and reduce material waste [37]. Real-time monitoring systems equipped with AI can detect and correct deviations from desired printing conditions, ensuring high precision and repeatability in the production of 3D-printed foods [150]. Additionally, AI-driven feedback mechanisms enable continuous improvement of printing processes by learning from previous prints and adjusting settings accordingly [39,151]. The application of AI in personalized nutrition leverages big data and ML to create customized dietary solutions [148]. By analyzing individual health data, dietary habits, and nutritional needs, AI can design 3D-printed foods that cater to specific health conditions, such as diabetes, hypertension, or malnutrition [152]. This level of customization not only enhances the nutritional value of food but also promotes better health outcomes by providing tailored nutrition [30,40]. Furthermore, AI enhances the sustainability of 3D food printing by optimizing the use of raw materials and minimizing waste. Intelligent design algorithms can create complex food structures that use minimal resources while maximizing nutritional content and sensory appeal [30,147]. AI also facilitates the use of alternative protein sources, such as plant-based or insect-based proteins [59], which can be precisely incorporated into 3D-printed foods to meet dietary requirements and sustainability goals [26].

Practically, artificial intelligence (AI) has been widely applied in food inspection and shelf-life prediction, demonstrating significant advancements in accuracy and efficiency across various food types [40]. For bananas, AI models such as Convolutional Neural Networks (CNN) and AlexNet have been utilized to predict fruit maturity based on color, achieving high accuracy rates of 98.25 %, 99.36 %, 81.96 % for CNN, and 81.75 %, 99.36 %, 81.75 % for AlexNet [153]. In the case of chicken meat, Genetic Algorithm (GA) and Artificial Neural Networks (ANN) are employed to predict freshness through color analysis [154]. Egg freshness is monitored using the Haugh unit with Machine Learning (ML) models and Near Infrared (NIR) spectroscopy, reaching an accuracy of 87.0 % [155]. For fish, AI models like Support Vector Machine (SVM) and CNN predict quality using Total Viable Counts (TVC) from images, achieving over 86 % accuracy [156]. Additionally, Partial Least Squares Regression (PLSR) and Feed-forward Neural Networks (FNN) determine fish freshness by analyzing TVB-N and TBA with visible and NIR images [157]. In frozen foods, Back-propagation ANN (BP-ANN) assesses quality based on drip loss and texture parameters using NIR spectroscopy [158]. Kiwifruit postharvest life is determined by nutrients using ANN and firmness measurements [159]. For Korean cabbage kimchi, various AI models including Multivariable Linear Regression (MLR), Random Forest (RF), and XGBoost predict quality by evaluating TLAB, pH, TA, and RSC [152]. These studies illustrate the extensive application and high accuracy of AI in enhancing food inspection and shelf-life prediction processes.

## 8 Hygienic requirements and food ink characteristic

Effective cleaning and sanitation protocols in food industries are crucial for maintaining hygiene standards [32,[160], [161], [162], [163]]. This involves specific cleaning and sanitization protocols, including the use of chemicals such as quaternary ammonium compounds, chlorine-based solutions, and hydrogen peroxide. Regular cleaning schedules should be implemented, with daily cleaning of removable parts, weekly deep cleaning of the entire system, and monthly maintenance checks. Methods for validating cleanliness include ATP bioluminescence testing, microbiological swabbing, and visual inspections [152,164,165]. These protocols involve the removal of soil from surfaces and the elimination of pathogenic microorganisms while not compromising product quality [166]. Chemicals like warm water, dish soap, and baking soda are commonly used for cleaning, with validation methods including CFU and PFU testing [161]. Different food industries implement specific cleaning and disinfection programs tailored to their needs, with verification activities ensuring compliance and continuous improvement [167]. These established protocols can serve as a foundation for developing hygiene standards in emerging technologies like 3D food printing, where similar principles of cleaning, sanitization, and validation can be adapted to ensure food safety and quality [168,169].

Comparing hygiene standards across different food industries can provide valuable insights. The dairy industry emphasizes cold chain management and rigorous cleaning with alkaline and acid detergents, practices that can be adapted for 3D food printing by integrating temperature control systems [26]. The meat industry utilizes UV sterilization and antimicrobial coatings to prevent microbial contamination, techniques that can enhance hygiene in 3D food printing setups [150,163]. The pharmaceutical industry's [8] use of HEPA filters and sterile packaging can also be mirrored in 3D food printing to prevent contamination [151]. Traditional food processing employs HACCP systems and detailed Sanitation Standard Operating Procedures (SSOPs) to manage hygiene risks systematically, which can be beneficial in 3D food printing [37,161,170]. Successful implementations in 3D food printing setups, such as a bakery integrating rigorous cleaning protocols and real-time AI monitoring, have significantly reduced microbial contamination [147,150]. Current regulatory standards from the FDA and EFSA emphasize equipment sanitation, proper food handling, and regular microbial testing, applicable to 3D food printing [30,161,170,171]. Interdisciplinary collaboration, incorporating insights from microbiology, food science, materials science, and engineering, is essential for developing effective hygiene practices [147]. Moreover, Ensuring personal hygiene and proper training for personnel involved in 3D food printing is vital [161,172]. More, hygiene recommendation can be summarized as follows:

Contamination Prevention: Preventing contamination during the 3D food printing process involves several strategies. One approach is the use of enclosed printing environments that minimize exposure to contaminants. Additionally, using disposable or easily sanitized components can further reduce the risk of contamination. Regular testing for microbial presence in food inks and printed products is also recommended to ensure hygiene standards are met. Real-time monitoring and rapid detection of contaminants using advanced sensors and AI technology are crucial [151].

Moreover, from the perspective of buyer, safety and less maintenance are considered important in choosing 3d printer. The design of 3D printer parts must be allowed to be disassembled and easily be cleaned [32]. The material of the printer parts that have direct contact with the food must be food certified [13]. If the extruder part is made of plastic, there is a risk of contamination [13,47]. Even if it is washed, there is still micro bacteria that remain in the piston, tubes, or extruder, and this cause the shelf life of the printed food short [49]. As suggested by Antonietta Baiano, it is better to cover the 3D printer parts with food grade polyurethane resins or epoxy [32]. Specifically for meat, it is required to keep the processing temperature below 4 °C to impede microbial growth [4]. Using better material such as stainless steel may prevent microbial contamination [49]. To date, there was no standard safety imposed by the government for 3D printed food, but international regulations of food, such as Codex Alimentarius, Sanitary and Phytosanitary Measures (SPS), and FAOLEX, can be inferred for guidelines [173].

For extrusion-based, there are 2 important characteristics, which are shear modulus, G*, and yield stress, YS [88]. The G* is a measure of the ability of the material to be deposited [16,82,88]. The equation of G* is shown in [8, 24].

(1) $$G^{*}=G^{\prime }+iG^{\prime \prime }\text{,}$$

where *G″* is the shear loss modulus, and *G′* is the shear storage modulus. If *G” > G′*, the material is suitable to be printed. The YS has two types, static and dynamic. The static shows the ability of the material to flow from rest, while the dynamic indicate its ability to resist high shear rates and its formability [14,88] …/.+.

### 8.1 Categories of food ink and hygiene

In 3D food printing, the selection and preparation of food inks are critical for achieving desired textures, flavors, nutritional profiles, and structural integrity of printed food products. Food inks must possess suitable rheological properties to ensure printability and stability during the printing process. Various categories of food inks have been explored, each offering unique properties and applications.

Some researchers classify three categories of food material: alternative ingredients, non-printable traditional materials, and natively printable materials [32]. Natively printable materials are food that are easily printed without manipulating the properties of the food [4]. Non-printable traditional materials are food that need additives/flow enhancers to be successfully printed [32]. Food materials that are unusual are classified as alternative ingredients, such as protein-fortified food ink [10], and these materials may be used to regulate the nutrients of the food [4].

**Safe Materials:** The selection of safe and food-grade materials for 3D printing is crucial. Food inks must be free from harmful additives and contaminants. Materials used in the construction of 3D printers, such as plastics and metals, should be certified for food contact to avoid chemical leaching. Using edible materials and ensuring regulatory compliance are essential in ensuring the safety of 3D-printed foods [76,174].

1. Dairy-Based Inks: When printing with dairy-based inks, maintaining cold chain logistics is essential to prevent spoilage and bacterial growth. The use of AI to monitor temperature and humidity levels during storage and transportation of dairy-based food inks is crucial [47,152,169].
2. Plant-Based Inks: For plant-based inks, such as those derived from vegetables and fruits, ensuring the removal of soil and pesticide residues is critical. Washing and pre-treatment of raw materials enhance the safety of plant-based 3D food inks ([59,68,76,76,95].
3. Meat-Based Inks: The use of meat-based inks requires stringent control of microbial contamination. Implementing UV sterilization and the use of antimicrobial coatings on printing surfaces can enhance hygiene in meat-based 3D food printing (Park et al., 2023) [4,50,68,106,119,140,150].
4. Insect-Based Inks: Insect-based inks present unique challenges due to their potential allergenicity and microbial load. Thorough processing and pasteurization of insect proteins ensure their safety as food inks ([26,76,106,175].
5. Hydrocolloid-based inks, such as gelatin, agar, carrageenan, xanthan gum (XG), and alginate, provide necessary viscosity and structural integrity, enhancing the flow properties and shape retention of gels. For example, XG improves the printability and shape fidelity of potato starch gels, making them suitable for extrusion printing. Carrageenan combined with soy protein enhances the printability and texture of oat-based pastes, while agar and gelatin are excellent for forming stable matrices in complex shapes [43,162,163,176].
6. Starch-based inks from potatoes, rice, corn, and wheat, often modified with hydrocolloids or proteins, improve printability. The incorporation of soybean polysaccharides into glutinous rice flour significantly enhances the retrogradation and gelatinization properties, reducing hardness and adhesiveness. Potato starch combined with XG and locust bean gum results in better fluidity and mechanical strength, ideal for detailed food structures [19,20,140,177,178].
7. Protein-based inks, such as soy protein isolate, whey protein, and casein, are used for their gelation, emulsification, and water-binding capacities. Soy protein isolated with oat flour forms a dense, extensible gel system, improving structural integrity. Whey protein with potato starch enhances storage modulus and viscosity, resulting in stable and self-supporting structures. Casein in cassava starch gels decreases the short-range ordered structure, enhancing elastic modulus and printing precision [21,82,102].
8. Fat and oil-based inks, like butter, cocoa butter, and oleogels, create rich, flavorful products. Butter powder added to mashed potatoes improves surface texture and printability, producing attractive and nutritious 3D printed foods. Beeswax-based oleogels used with potato starch-whey protein systems enhance printing accuracy and performance, yielding detailed and high-quality food items [22,27,179].
9. Vegetable and fruit purees, including those from carrots, tomatoes, and berries, offer natural, nutritious, and colorful inks for health-enhancing products. Mashed potatoes combined with strawberry juice gel result in multi-flavor, multi-texture foods that are visually appealing and nutritionally balanced. Microalgae-rich batters used in bakery products enhance sensory properties and nutritional profiles [13,41,110].
10. Insect-based inks, such as cricket powder, provide high-quality protein and essential nutrients. When combined with materials like mashed potatoes or soy protein, these inks improve nutritional value and printability, addressing global food security and sustainability challenges [180].
11. Customized nutrient-rich inks, formulated with bioactive ingredients [181], vitamins, and minerals, address specific dietary needs and health conditions. For example, curcumin nanoemulsion-filled gels embedded into 3D printed products enhance bioavailability, providing antioxidant and anti-inflammatory benefits. Carrageenan-based inks produce low-calorie surimi, making them suitable for the elderly [71,132].

The development and application of diverse food inks enable the creation of personalized, nutritious, and aesthetically pleasing food products, driving innovation in 3D food printing technology.

### 8.2 Additives/flow enhancers

By adding additives, the printability of the food ink is enhanced [12,14,29]. For meat products, transglutaminase or gelatin may be used [4]. By adding transglutaminase, the deposited food have better rigidity because of the self-supporting hydrogels [12,43,49]. Pectin also improves the consistency of the fruit-based ink [46]. Common additives for plant-based are xanthan gum, agar-agar, alginate, glycerol, lecithin, methylcellulose, and starch [20,46,49,110]. By adding additives, the dimensional stability may be improved [55]. In any case, it is recommended for the food ink to have shear-thinning properties [9,32].

## 9 Challenge and limitation

Even though the advancement of foods printing technologies has noticeably increased, however some of the challenges and limitations of food 3D printing should be addressed. Food 3D printing is yet inadequate way for massive food production, since it is a slow manufacturing process, and has high production costs (for large quantities). As a result, few have engaged food printing in real-life scenario. Consequently, food 3D printing technology is typically used in prototyping, research, or in special cases like food for space. Furthermore, varied recipe mixing methods are still an ongoing concern in food 3D printing. Likewise, researchers and developers must consider the various material characteristics of food as well as dietary quality. Eventually, from the previous sections we summarize different ongoing technical limitation as follows.

1. Although some people are open to food 3D printing, there were still others who were reluctant to try food 3D printing. Therefore, it is necessary to improve some components of the food 3D printing for costumers to embrace. For example: increasing safety, simplified 3D printing process, better shelf life of ink, etc.
2. In extrusion-based technique, only screw-based and gear-based have continuous process, and both have their own limitation. Therefore, new printers imposed new design for continuous process and without the drawbacks of screw-based and gear-based mechanisms.
3. By bioprinting cultured meat, couples of problems may be resolved, but the process of bioprinting for now is more expensive than traditional method. Therefore, a search of economical and safe materials for bioprinting process is the key for market adoption.
4. Although some of the materials do not need an additive to be printed, some researchers still used it. The printing parameters also changed with each material, which made things more complicated. Therefore, natural additives and a simple open cloud storage for saving printing parameters may be implemented.
5. A need for international standard for food 3D printers' company to meet safety standard of printed food.
6. Future regulatory frameworks should address the unique challenges of 3D food printing, such as the use of novel food inks and complex printing structures. Potential guidelines could include standards for the development and testing of food-grade materials, the implementation of AI-driven monitoring systems, and protocols for the safe disposal of waste materials. Collaborative efforts between regulatory bodies, industry stakeholders, and researchers will be essential in developing these frameworks [76]. Using eco-friendly cleaning agents and sustainable materials for equipment construction, along with methods for managing waste generated from cleaning processes, can enhance the sustainability of 3D food printing [174].

## 10 Conclusion

Customized food printing is becoming rapidly popular among the many uses of 3D printing. Foods, such as meat, chocolate, candy, pizza, cookies, and bread, can be manufactured layer-by-layer with 3D food printing technology while controlling the food's nutrient content, amount, and taste. As shown in.

Table 3 and Table, globally, research efforts have been made by companies, research institutes, and researchers in order to achieve a distinctive approach and develop food printing technologies and printable food (food ink) in the worldwide industry. Most of the printing method/technique is using extrusion-based method due to its versatility. However, even with it, only some types of food, for now, can be 3D printed. Although progress has been made, food 3D printing is still not suitable for mass products, but this is not a serious problem, since food printing is for customization. There is also a specific parameter for each type of food that may complicate the printing process, including the need for additives. There is still a need to make a better extrusion process that can be used for a wide range of materials and that is continuous. Shortly, food 3D printing has many benefits to give, but in the implement stages, there are still a need for improvements.

## Data availability

No data was used for the research described in the article.

## CRediT authorship contribution statement

**Husam A. Neamah:** Writing – review & editing, Supervision, Conceptualization. **Joseph Tandio:** Writing – original draft, Formal analysis.

## Declaration of competing interest

The authors have no relevant financial or non-financial interests to disclose.

Please address all correspondence concerning this manuscript to me at [ husam@eng.unideb.hu ].
